# Light Induced Regulation Pathway of Anthocyanin Biosynthesis in Plants

**DOI:** 10.3390/ijms222011116

**Published:** 2021-10-15

**Authors:** Yanyun Ma, Xu Ma, Xiang Gao, Weilin Wu, Bo Zhou

**Affiliations:** 1 Key Laboratory of Saline-Alkali Vegetation Ecology Restoration, Northeast Forestry University, Ministry of Education, Harbin 150040, China; myy060528@163.com (Y.M.); maxu990620@163.com (X.M.); 2 College of Life Science, Northeast Forestry University, Harbin 150040, China; 3 Key Laboratory of Molecular Epigenetics of MOE and Institute of Genetics & Cytology, Northeast Normal University, Changchun 130024, China; gaox891@nenu.edu.cn; 4 Agricultural College, Yanbian University, Yanji 133002, China

**Keywords:** anthocyanin biosynthesis, transcription factors, environmental regulation, light signal, regulatory network

## Abstract

Anthocyanins are natural pigments with antioxidant effects that exist in various fruits and vegetables. The accumulation of anthocyanins is induced by environmental signals and regulated by transcription factors in plants. Numerous evidence has indicated that among the environmental factors, light is one of the most signal regulatory factors involved in the anthocyanin biosynthesis pathway. However, the signal transduction of light and molecular regulation of anthocyanin synthesis remains to be explored. Here, we focus on the research progress of signal transduction factors for positive and negative regulation in light-dependent and light-independent anthocyanin biosynthesis. In particular, we will discuss light-induced regulatory pathways and related specific regulators of anthocyanin biosynthesis in plants. In addition, an integrated regulatory network of anthocyanin biosynthesis controlled by transcription factors is discussed based on the significant progress.

## 1. Introduction

Plant growth and development are greatly affected by the environment. Anthocyanin production is affected by various environmental factors such as light, low temperature, drought, and salinity [1,2,3]. Light is a particularly important environmental factor inducing anthocyanin accumulation in plants. Different types of anthocyanin are widely distributed in various plant tissues such as flowers, fruits, stems, leaves, and underground tubers [4]. The anthocyanin accumulation in these plant tissues is shown as light-dependent or light-independent biosynthesis. The different light quality and intensity are perceived by receptors or signal factors to transduce them into downstream transcription factors [5]. Next, metabolites are synthesized by enzymes encoded by structural genes that are regulated by transcription factors to respond to light. Anthocyanins belonging to the flavonoids are important secondary metabolites to adapt to harmful environmental stress in plants.

Anthocyanins are universal water-soluble flavonoid pigments that are responsible for the widest color of leaves, petals, fruits, seeds, stems, and tubers of plants (Liu et al., 2021) [6]. Moreover, they have been shown to play a beneficial role in the visual activity, cancer, heart disease, and age-related neurodegenerative disorders [7], although no effects or even a negative effect on health-related parameters have also been reported [6,8]. Anthocyanins consist of an anthocyanidin backbone with sugar and acyl conjugates while anthocyanidins are composed of two aromatic benzene rings and an oxygenated heterocycle [9,10]. Due to the number of hydroxyl groups and glycosyl groups in the rings, anthocyanins have potent antioxidant activity to scavenge free radicals and reactive oxygen species (ROS) during environmental stresses [6]. Therefore, anthocyanins have protective effects during plant development through absorbing excess UV light, preventing lipid peroxidation and suppressing the activity of ROS. Then, plants have evolved to the biosynthetic pathways of anthocyanins to resist various abiotic stresses including UV irradiation, drought, high salinity, and low temperature [11].

Recently, a great number of studies have revealed that anthocyanin accumulated when plants are under environmental stress. In *Arabidopsis*, the accumulation of photoprotective anthocyanin pigments is light-dependent [12], which improves salt resistance under salt stress [13]. In Chinese bayberry fruit, blue light can enhance anthocyanin accumulation and improve the commercial and nutritional value of Chinese bayberry fruit [14]. In addition, the anthocyanin of ‘Red Globe’ in grape is a typical light-dependent accumulation, whose anthocyanin synthesis in the berry skin is induced by light [15]. In red apples, anthocyanin biosynthesis is also regulated by the light-induced expression of MdMYB1 [16]. Moreover, abiotic stress-induced anthocyanin accumulation and UVR8 (UV RESISTANCE LOCUS 8) expression are also indicated to be light-dependent and both H_2_O_2_ and NO are involved in UV-B-induced anthocyanin accumulation in radish sprouts [17]. Next, the pathway of light induces signal factors to activate the transcription of anthocyanin biosynthesis genes to produce anthocyanins for stress tolerance is concluded. However, many signal transduction regulators and key transcription factors in the complicated anthocyanin biosynthesis regulatory network remain to be identified. To provide a detailed overview of the known molecular regulatory mechanisms of light-dependent and -independent anthocyanin biosynthesis in plants and provide a theoretical foundation for the application of colored photo-selective nets in the breeding of crops, fruits, and ornamental plant species, this review focuses on known physiological, biochemical, and molecular mechanisms involved in the light regulation of anthocyanin biosynthesis and their role in abiotic stress tolerance.

## 2. Anthocyanins Accumulation in Plants

**Figure 1:**
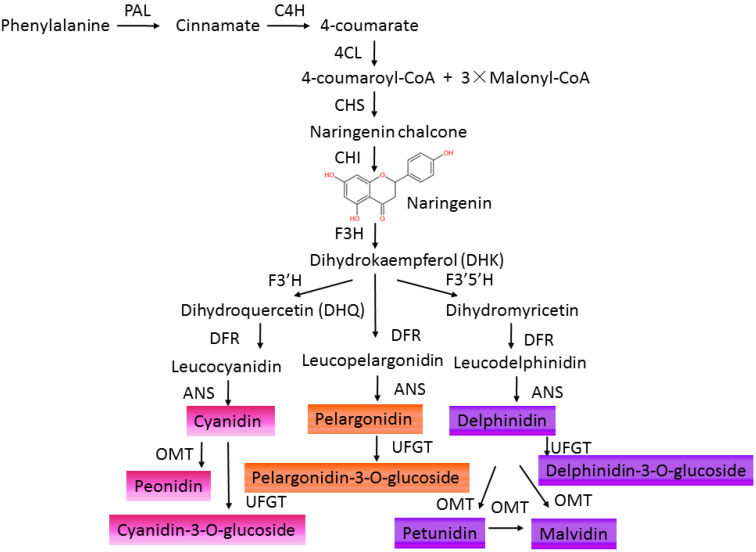
The pathway of anthocyanin biosynthesis. PAL, phenylalanine ammonia lyase; C4H, cinnamate 4-hydroxylase; 4CL, 4-coumarate CoA ligase; CHS, chalcone synthase; CHI, chalcone isomerase; F3H, flavanone 3-hydroxylase; F3′H, flavonoid 30 hydroxylase; F3′5′H, flavonoid 3050hydroxylase; FLS, flavonol synthase; DFR, dihydroflavonol 4-reductase; ANS, anthocyanidin synthase; UFGT, UDP-galactose flavonoid 3-O-galactosyltransferase; OMT, O-methyl transferase.

In the natural environment, anthocyanins participate in the formation of multicolors in fruits, flowers, leaves, even roots of plants [7,18]. Different kinds of anthocyanins modified by methylation and hydroxylation at different positions of C6-C3-C6 carbon skeleton structures decide different colors varying from orange, red, and purple to blue in plants [7,19] (Table 1). The physiological role of anthocyanin in plants consists of defending against viral, bacterial, and fungicidal activities, absorbing excess visible and UV light irradiance, attracting pollinators and seed dispersers, and scavenging excess ROS under abiotic stresses [6,11,20]. Anthocyanins are synthesized in the endoplasmic reticulum and transported to accumulate in the vacuoles of a wide range of cells and tissues in both the vegetative and reproductive organs of plants [20]. The synthesis of anthocyanins in plants is controlled by structural genes and can be divided into four stages [21,22]. The early stage is from phenylalanine to 4-coumaryl CoA sequentially catalyzed by phenylalanine ammonialyase (PAL), cinnamate 4-hydroxylase (C4H), and 4-coumarate-CoA ligase (4CL). Then, 4-coumaroyl CoA is converted to dihydrokaempferol (DHK), which is regulated by chalcone synthase (CHS), chalcone isomerase (CHI), and flavanone 3-hydroxylase (F3H). DHK can also be converted to dihydroquercetin (DHQ) by flavonoid 3′-hydroxylase (F3′H) or catalyzed by flavonoid 3′5′-hydroxylase (F3′5′H) to form dihydromyricetin. Next, DHK, DHQ, and dihydromyricetin are separately converted to leucopelargonidin, leucocyanidin, and leucodelphinidin by dihydroflavonol 4-reductase (DFR). Finally, these leucoanthocyanidins are catalyzed by anthocyanidin synthase/leucoanthocyanidin dioxygenase (ANS/LDOX) to form colored anthocyanidins (pelargonidin, cyanidin, delphinidin) [23]. Then, these anthocyanidins can be further modified by UDP-glucose flavonoid glucosyltransferase (UFGT), acetylase, O-methyltransferase (OMT), and anthocyanin transferase (AT) [24] to produce stable and water-soluble pigments (Figure 1). These flavonoid products vary in different plants due to the evolution of anthocyanin metabolism under environmental selective pressures [25]. Anthocyanins show potent antioxidant activities and are effective scavengers of ROS in vitro [26,27]. In addition, anthocyanins are involved in regulating ROS-induced signaling responding to environmental cues [28,29,30]. Anthocyanins may be regulators of the ROS-signaling network due to their ability to interact with protein and to enhance the activity of protein through the ROS reaction [20,31,32]. However, the functional roles of flavonoids in plants remain indistinct not only for their numerous varieties, but also for their different influences in different plant species. According to different properties of solubility, light absorption, and distribution patterns in various parts of the plant, anthocyanins have been cataloged numerically [33], and the composition profile of anthocyanins varied under different stresses [34,35]. The biological functions of different types of anthocyanins still need to be identified in various plants. Moreover, the roles of family genes, encoded enzymes of the anthocyanin biosynthetic pathway, also need to be clarified [36,37,38].

## 3. Regulatory Pathway of Anthocyanin Biosynthesis

### 3.1. Regulatory Factors Involved in Anthocyanin Biosynthesis

According to the research of *Arabidopsis*, it is well known that early anthocyanin biosynthetic genes (*CHS*, *CHI*, *F3H*, *F3′H*, and *FLS*) are regulated by R2R3-MYB transcription factors MYB11, MYB12, MYB111, and MYB75 while the late biosynthetic genes (*DFR*, *ANS*/*LDOX*, *UFGT*) are regulated by the MBW complex (R2R3-MYBs such as MYB75, MYB90, MYB113, and MYB114, bHLH, and WD40) [39,40,41]. In addition to MYB-bHLH-WDR transcription factors, other regulatory proteins have also been identified to be involved in anthocyanin biosynthesis in plants including members of the NAC transcription family [42,43], MADS-box proteins [44,45,46], bZIP transcription factors [47,48,49], WRKY transcription factors [50], and SPL transcription factors [51,52]. These transcription factors take positive or negative roles in regulating the expression of structural anthocyanin biosynthesis genes and determine the anthocyanin accumulation in developmental and environmental regulation.

### 3.2. The Activation of Positive R2R3-MYB Factors Is Sufficient for Promoting Anthocyanin Biosynthesis

Among these transcription factors, MYB transcription factors play a significant role in regulating anthocyanin biosynthesis. Many anthocyanin-related R2R3-MYB factors have been identified from many plants such as *Arabidopsis* [39], grape [53], Gerbera [54], eggplant [55], tomato [55], populous [56], apple [57,58], etc. Most of these are R2R3-MYB proteins with the motif [D/E]LX2[R/K]X3LX6LX3R in the R3 domain, which is necessary to interact with bHLH acting as activators to increase anthocyanin accumulation [59]. In *Arabidopsis*, MYB75 (PAP1), MYB90 (PAP2), MYB113 (PAP3), and MYB114 (PAP4) show very high conserved sequences, and their overexpression increases the accumulation of anthocyanin through TTG1- and bHLH-dependent regulating late anthocyanin pathway genes [39]. In addition, the transcription level of PAP1 is higher than that of the other three MYB genes in young seedlings of *Arabidopsis*. In *AtMYB75* transgenic tomato plants, anthocyanin production was also induced in tomato plants [60]. Moreover, overexpressing a single SlMYB75 TF can lead to abundant anthocyanin accumulation in both vegetative and reproductive organs of tomatoes [61] and another MYB TF SlAN2-like was also identified to be responsible for the *Aft* phenotype (anthocyanin fruit). In blueberry, VcMYBL1 has been identified to interact with VcbHLHL1 to promote anthocyanin biosynthesis [62]. In apple, MdMYB3 is also characterized to activate transcriptional flavonoid pathway genes and regulate the accumulation of anthocyanin in the skin of apple fruits [63]. SmMYB1 isolated in eggplant can interact with a heterologous bHLH, but the C-terminal domain in SmMYB1 is essential for transcriptional activation of anthocyanin genes [64].

### 3.3. The Varieties of R2R3 MYBs Determine the Regulatory Specificity of Anthocyanin Accumulation

These R2R3 MYBs have conserved R2R3 domain sequences, which determine anthocyanin pathway specificity. For example, in *Arabidopsis*, PAP4 activates the transcription of *UFGT*, which can produce anthocyanins from anthocyanidins, while TT2 regulates the expression of *ANR*, which is specific to proanthocyanin biosynthesis [65]. Alteration of individual amino acids of the R2R3 domain in PAP4 and TT2 can lead to changes in activation of the *ANR* and *UFGT* promoter. The amino-acid change in TT2 (Gly^39^ →Arg) can switch TT2 specificity toward the anthocyanin pathway while the amino-acid mutant (Arg^39^ → Gly) and exchange of motif (Ala-Asn-Asp-Val → Asp-Asn-Glu-Ile^90−93^) in PAP4 switches PAP4 specificity toward the PA pathway [65]. The orthologs VvMYBA2 and VvMYBPA2 in grapevine also have similar amino acids for pathway specificity [65]. Therefore, the anthocyanin pathway specificity is determined by the amino acid composition of the R2R3 domain in MYB transcription factors and the cis-elements in the promoters of anthocyanin biosynthetic genes. Due to the conservation of R2R3-MYB, regulatory differences are determined by promoter divergence rather than the divergence of the regulators [65,66]. Then, it is not difficult to understand that MYB transcription factors particularly regulate the expression of different anthocyanin pathway genes in various plants. In *Arabidopsis*, MYB11, MYB12, and MYB111 activate promoters of early genes (*CHS*, *CHI*, *F3H*, and *FLS*) but not of late genes (*F3′H* and *DFR*) [67,68]. However, the expression of *PAL1* and *CHS* remains unchanged in MYB75, MYB90, MYB113, and MYB114 RNAi seedlings, and the transcription for the late genes *DFR*, *F3′H*, *UGT75C1*, *LDOX*, and *GST12* is reduced [39]. In apple fruit, MdMYB114 also promotes anthocyanin accumulation by directly controlling the expression of late genes *MdANS*, *MdUFGT*, and *MdGST* [58], but in turnip (*Brassica rapa*), BrMYB75 can bind the promoter of early gene *CHS* to regulate anthocyanin biosynthesis [36]. Moreover, overexpression of MdMYB90-like in apple bud can induce the expression of both early and late genes including *MdCHS*, *MdCHI*, *MdANS*, and *MdUFGT* [57]. In tomatoes, two anthocyanin-related R2R3-MYB factors take different tissue-specific regulatory roles in anthocyanin accumulation. One is SlAN2/SlMYB75, which is as a positive regulator of anthocyanin biosynthesis in vegetative tissues of tomato plants including hypocotyls, cotyledons, stems, and leaves [69], and the other is SlAN2-like/Aft, which determines anthocyanins in the fruits of tomato plants [70,71].

### 3.4. The Negative MYB Proteins Repress Overexpression of Anthocyanin Biosynthetic Genes to Maintain the Balance of Pigment Accumulation

In contrast, some MYB proteins play a negative role in regulating anthocyanin biosynthesis such as AtMYB4, AtMYB7, AtMYBL2, AtCPC in *Arabidopsis* [72,73], FaMYB1 in strawberry [74], VvMYBC2-L1 in grapevine [75], and SlTRY, SlMYBATV in tomato [76,77]. AtMYB4 and AtMYB7, with the C-terminal EAR (ERF-associated amphiphilic repression) transcription repressor motif (with LxLxL or DLNxxP core sequence), repress the flavonoid biosynthesis by negatively regulating *DFR* and *UGT* gene expression [78], while AtMYBL2 with the C2 repressor motif (pdLNLD/ELxiG/S) can suppress the expression of *DFR* and *TT8* to regulate anthocyanin biosynthesis [73]. Moreover, AtCPC, which is just like AtMYBL2 with single-repeat R3, acts as competitive inhibition of the MBW complex to negatively regulate the anthocyanin pathway in *Arabidopsis* [79]. In grape berry, the R2R3-MYB protein VvMYBC2-L1 as AtMYB4 has also conserved the C2 motif in the C-terminal region and acts as a direct repressor to downregulate the expression of phenylpropanoid synthesis genes or as an indirect repressor to compete with MYB activator via binding affinity with the promoter of bHLH and WD40 [80]. The R2R3-MYB repressor FaMYB1 with the DNEV motif in strawberry is different to the AtMYB4 motif DNEI and the different motifs in the C-terminus of AtMYB4-like and FaMYB1-like repressors show potentially distinct mechanisms of action. SlTRY, SlMYBATV in tomato such as AtCPC, and AtMYBL2 belong to single-repeat R3-MYB factors without a repressive motif, but they retain the motif responsible for binding to bHLH. The role of these MYB repressors in anthocyanin biosynthesis has been reviewed in detail [81,82]. Therefore, the regulation of MYB repressors and activators maintains the balance of accumulation of anthocyanin in plants, and MYB transcription factors are considered key components that provide specificity for the downstream genes and cause tissue-specific anthocyanin accumulation [83].

### 3.5. Different bHLH Proteins Interact with MYB Factors to Form Specific MBW Complex

As cofactors of MYB activators and repressors, bHLH proteins incorporating the MYB-interacting region, WD40/AD domain, basic helix–loop–helix domain, and ACT domain are responsible for MYB interaction and promoter binding of anthocyanin biosynthetic genes [84]. The bHLH family has been divided into 26 subfamilies [85] and the IIIf subfamily bHLH members are involved in both flavonoid biosynthesis and trichome formation [86]. The binding of the bHLH proteins with WD40 protein can be modulated by different R2R3 MYB proteins. In *Arabidopsis*, three bHLH TFs, GL3, EGL3, and TT8, have been shown to interact with R2R3-MYB proteins and regulate flavonoid biosynthesis [39,87]. AtTT8 can interact with AtTTG1 to form stable MBW complexes with different R2R3 MYBs while AtGL3 and AtEGL3 show competitive complex formation with some R2R3 MYBs [88]. The AtTTG1–bHLH interactions are modulated positively and negatively through the addition of R2R3 MYB proteins in *Arabidopsis*, [88]. PhAN1 and PhJAF13 in petunia have also been identified to be involved in anthocyanin [89,90]. In Solanaceous plants, AN1 directly regulates the expression of biosynthetic genes, whereas JAF13 can regulate the transcription of AN1 [91]. Then, the IIIf subfamily bHLHs can also be further divided into JAF13 clade and AN1 clade [90]. In the petunia hybrid, the interaction of PhAN1 with PhAN11 is promoted by the addition of the phAN4 R2R3 MYB protein, while the interaction of PhJAF13 with PhAN11 is significantly reduced in the presence of R2R3 MYB proteins [88]. Therefore, in different species, even in different tissues of the same plant, the functions of the bHLHs and MBW complexes vary. For example, AcMYB123 and AcbHLH42 can activate the function of the promoters of *AcF3GT1* and *AcANS* and be involved in the spatiotemporal regulation of anthocyanin biosynthesis, specifically in the inner pericarp of kiwifruit [92]. MdbHLH3 can also bind to the promoters of *MdDFR*, *MdUFGT*, and *MdMYB1* to activate their expression, which is involved in anthocyanin biosynthesis in apples. The individual combinations of bHLH and R2R3 MYB proteins regulate the expression of different anthocyanin synthetic genes and determine the spatiotemporal pigment accumulation in plants.

### 3.6. WD40 Protein, the Stabilizer of MBW Complex

The WD40 protein has 4–10 random WD repeat domains, which consist of 40 amino acid sequences ending in tryptophan (W) and aspartic acid (D). In *Arabidopsis*, AtTTG1, a member of the WD 40 proteins, is related to seedling anthocyanin accumulation and seed coat pigmentation [93]. AtTTG1-GL3/EGL3/TT8-TT2/MYB5 complexes are responsible for seed while AtTTG1-GL3/EGL3/TT8-PAP are relevant in vegetative tissue [94,95]. MdTTG1, the WD40 protein in apple, can interact with MdbHLH3 and MdMYB9 to control the expression of downstream structural genes [96]. Among the MBW complexes, WD40, which is involved in stabilizing the MBW complex, is generally similar between anthocyanin-pigmented and non-pigmented tissues [97,98].

### 3.7. Other Regulation Proteins beyond MBW Complex

**Figure 2:**
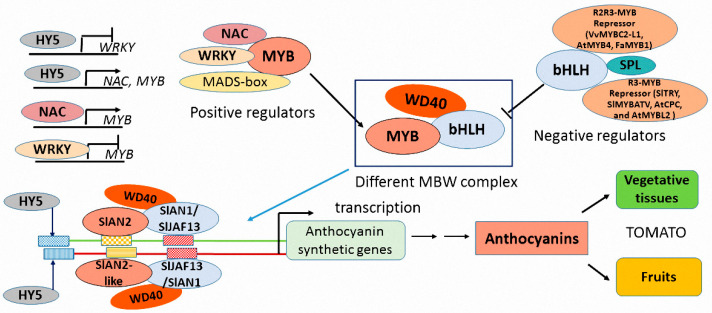
The regulatory pathway of anthocyanin biosynthesis in plants. NAC, WRKY, MADS-box can interact with MYB to form BMW complex and positively regulate the expression of anthocyanin synthetic genes. In the regulatory pathway, the expression of *NAC*, *MYB* is regulated by HY5, NAC, and WRKY, and the expression of *WRKY* is also regulated by HY5. However, some negative regulators such as VvMYBC2-L1, AtMYB4, FaMYB1 belonging to R2R3-MYBs, SlTRY, SlMYBATV, AtCPC, and AtMYBL2 belonging to R3-MYBs interact with bHLH to compete with MYB and disturb the MBW complex. Another negative regulator SPL can also interact with bHLH to affect MYB-bHLH-WD40 complex. The transcription factor HY5 and MBW complex which consists of different MYB proteins such as SlAN2 and SlAN2-like and bHLH proteins such as SlAN1 and SlJAF13 in tomato can bind to the promoters of different anthocyanin synthetic genes to activate their transcription and accumulate anthocyanin in specific vegetative tissue or fruits.

In addition to the MBW complex, the NAC, MADS, bZIP, WRKY, SPL transcription factors are also involved in the regulation of anthocyanin biosynthesis. A NAC transcription factor, MdNAC42, can interact with MdMYB10 to positively regulate anthocyanin accumulation in red-fleshed apples [43]. Moreover, MdHY5, a bZIP protein, promotes anthocyanin biosynthesis by positively regulating its own transcription and that of MdMYB10 and even downstream anthocyanin biosynthesis genes through binding to E-box and G-box motifs in apple [49]. MdHY5 can also promote the expression of MdNAC52, which regulates the expression of MdMYB9 and MdMYB11 to increase anthocyanin biosynthesis [46]. Another MdbZIP44, an ABA-induced bZIP transcription factor, can interact with MdMYB1 to enhance the binding of MdMYB1 to its downstream genes and promote anthocyanin accumulation [47]. MdWRKY40, a wounding-responsive protein in apple, has also been identified to interact with MdMYB1 and enhance the binding of MdMYB1 to its target genes for anthocyanin biosynthesis [99]. In *Arabidopsis*, a WRKY TF TTG2 can interact with the WD-repeat protein TTG1 in the MBW complex to form a four-component complex involved in the PA pathway [100,101]. However, AtWRKY41 represses anthocyanin accumulation by negatively regulating the expression of AtMYB75, AtMYB111, and AtMYBD in *Arabidopsis* [50]. As in apple, MdWRKY41 is negatively regulated by MdHY5, which weakens the effect of the MdWRKY41-MdMYB16 repressor on anthocyanin accumulation [102]. To VmTDR4, the MADS-box protein in bilberry regulates the accumulation of anthocyanins through direct or indirect control of R2R3 MYB transcription factors [45]. In addition, the FcMADS9 protein in fig promotes anthocyanin accumulation, and ethylene has been proven to be involved in its regulation [103]. Furthermore, the coloration regulation of SlMADS-RIN in tomato and MdMADS1 in apple is related to ethylene, which suggests a link between MADS-box transcription factors regulating anthocyanin accumulation and ethylene [104,105,106]. Conversely, SQUAMOSA PROMOTER BINDING PROTEIN-LIKE (SPL) genes targeted by miR156, take a negative role in anthocyanin accumulation through destabilization of an MYB-bHLH-WD40 transcriptional activation complex [52]. In a word, a large number of anthocyanin activators and repressors have been confirmed to be involved in regulating the expression of anthocyanin biosynthetic gene components and the stabilization of the MBW complex. Therefore, anthocyanin biosynthesis is positively or negatively regulated by multi-transcription factors through interacting with the MBW complex or directly binding to the promoters of anthocyanin biosynthetic genes (Figure 2).

## 4. Light Induced Anthocyanin Accumulation in Plants

### 4.1. Light Receptor and Light Signal Transduction

Light is essential for plant growth and development, but excess high-energy UV irradiance can cause damage to a cell, and anthocyanin accumulation in plants benefits plants in enhancing resistance to UV stress [20,107]. Plants have developed sophisticated photoreceptor systems such as phytochromes (red/far-red photoreceptors), cryptochromes, and phototropin (blue/UV-A photoreceptors) and UVR8 (UV-B photoreceptors) to adapt to variable light radiation [108]. Many plants accumulate anthocyanin in a light-dependent manner, that is, anthocyanin biosynthesis of these species is light induction. For example, the anthocyanin accumulation in fruits such as tomato [70], apple [16,109,110], pear (*Pyrus pyrifolia*) [111,112], lychee (*Litchi chinensis*) [113], and grape (*Vitis vinifera*) [114]. Photoreceptors sense the different light environments and bring about structural change or modification of the receptor protein. Then, the activated receptor proteins transfer to the nucleus and interact with positive transcription factors or inactivate a master negative regulator COP1 to regulate the expression of downstream genes [108].

Phytochromes have been reported to perceive a high red/far-red ratio to convert from the Pr to Pfr isoform, and the active Pfr form can interact with PIFs to regulate light-regulated gene expression. In addition, activated phytochromes can rearrange the COP1–SPA complex to make it non-functional or inactivate the COP1-SPA E3 ligase to avoid the degradation of positive transcription factors such as HY5 [115,116,117]. Moreover, the photoactivated cryptochrome oligomers interact with cryptochrome-interacting proteins such as COP1, CIB, SPA, BIC, PIF, PPK, AUX/IAA, AGB, and phytochrome to form a cryptochrome complexome mediating blue-light regulation of transcription or protein stability [118]. The CRY–COP1–SPA interaction positively regulates the abundance of the HY5 protein [119]. For the UV-B receptor, UVR8 protein absorbing UV-B light causes the UVR8 dimer to break and the resulting monomer migrates into the nucleus to interact with COP1 and form UVR8–COP1–SPA complexes [120,121,122]. The UVR8–COP1–SPA interaction also results in the stabilization of HY5 to initiate UV-B mediated gene expression. Natural UV light intensities and high temperatures have been reported to induce increased acylation levels of anthocyanidin (the delphinidin and petunidin derivatives) in *Vitis vinifera* [123], and the flavonol and anthocyanin accumulation is also impacted in the treatment of light quality/quantity (UV-B) and temperatures during the berry development of grape [124]. This implies that different light quality/quantity can affect the activity of anthocyanin biosynthetic genes to produce different anthocyanidin derivatives that are also regulated by other abiotic factors such as temperature and drought [125].

### 4.2. Light Signal Transduction Factor HY5

HY5, which is unstable in darkness and is degraded by COP1 via the 26S proteasome pathway or is light stable through SPA by dissociating with COP1 [126], has been identified to act as a master regulator of light signal transduction and directly activate the expression of anthocyanin biosynthesis and regulatory genes including MdMYB1 in apple [49] and AtPAP1/MYB75 in *Arabidopsis* [127]. HY5 can bind the T/G-box (CACGTT), E-box (CAATTG), GATA-box (GATGATA), ACE-box (ACGT), Z-box (ATACGGT), and C-box (GTCANN) as well as the hybrid C/G- (G) and C/A-boxes in the promoters of many genes that are involved in light signaling [128] and anthocyanin biosynthesis [127]. In tomatoes, the light-responsive SlAN2-like can activate the expression of both anthocyanin biosynthetic genes and their regulatory genes to accumulate anthocyanin. However, if a functional SlAN2-like gene is driven by the fruit-specific promoter in a tomato cultivar, the high-level anthocyanins will accumulate in both the peel and flesh with light-independent biosynthesis [70]. Then, a light signal is perceived by photoreceptors and transduced to HY5 and other regulated proteins to activate MYB transcription factors for light-dependent anthocyanin biosynthesis.

### 4.3. HY5-Dependent and -Independent Pathway Regulate Light-Dependent and -Independent Anthocyanin Biosynthesis Separately

However, when the MYB regulated proteins and anthocyanin biosynthetic genes are activated to express constitutively, the anthocyanin accumulation will display light-independent biosynthesis. The research of light in regulating anthocyanin biosynthesis in dark-red and bicolored cherry cultivars showed that light is necessary for anthocyanin biosynthesis in bicolored cherries, but not in the dark-red fruits [129]. In blueberry (Vaccinium spp.), the UV-B treatment induces HY5 expression to upregulate VcMYBPA1 and downregulate VcMYBC2, then promotes the accumulation of anthocyanins in the green fruit stage. Whereas in the mature fruit stage, anthocyanin synthesis is inhibited by increased VcMYBC2 levels when exposed to UV-B light through the HY5-independent pathway [130]. The stage-dependent anthocyanin biosynthesis in UV-B exposed blueberry is coordinately balanced by the anthocyanin-related MYB activators and repressors. Then, the light-dependent anthocyanin biosynthesis is regulated by ‘double-negative logic’ [82], that is, anthocyanin accumulation is determined by light-induced nuclear export or inactivation of the repressor COP1–SPA complex [131], de-repressing the HY5, and activating the MBW complex and anthocyanin biosynthesis. Certainly, other protein factors such as B-box (BBX) proteins can also respond to light and interact with HY5 to regulate the transcription of anthocyanin biosynthetic genes. In red pears, both PpBBX16 and PpBBX18 can form the PpBBX–PpHY5 complex to activate the expression of *PpMYB10* and regulate anthocyanin accumulation [111,132], but PpBBX21, a negative regulator, can hinder the formation of the PpBBX18–PpHY5 complex and repress anthocyanin biosynthesis [132]. Moreover, MdBBX22 promotes UV-B-induced anthocyanin biosynthesis by MdBBX22-MdHY5 interaction in apple, and the expression of *MdBT2* is suppressed by UV-B treatment which can degrade the MdBBX22 protein through the 26S proteasome pathway [133]. MdTCP46 and MdMYB1 also play positive roles in anthocyanin biosynthesis induced by high light intensity, but under low light intensity, MdBT2 ubiquitinates and degrades the MdTCP46 (Teosinte branched1/cycloidea/proliferating transcription factor 46) and MdMYB1 proteins to repress anthocyanin biosynthesis [110]. Furthermore, blue light signal transduction module CRY-COP1-HY5 contributes to the anthocyanin biosynthesis induced by blue light in red pear, but red light does not affect anthocyanin accumulation [112]. However, in ripening bilberry fruits, blue and red light are effective in inducing anthocyanin and delphinidin accumulation via CRY2/COP1 and HY5 or ABA-signal transduction [134]. Red light, blue light, and red/blue compound light also induce the strawberry fruit coloration [135]. Therefore, light intensity and light quality can induce different signal transduction factors involved in light-dependent anthocyanin biosynthesis.

**Figure 3:**
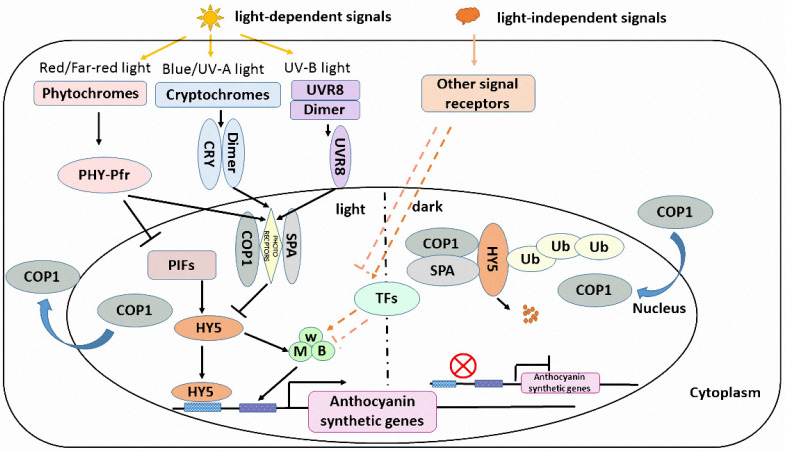
A simplified model of light-dependent and -independent regulation of anthocyanin biosynthesis in plants. During light-dependent anthocyanin biosynthesis, photoreceptors sense different wavelengths of light and are activated to be transferred into the nucleus, then the HY5-COP1-SPA complex is rearranged to dissociate HY5 protein and to avoid the degradation of HY5 transcription factors. HY5 positively regulates the expression of anthocyanin biosynthetic genes and lead to pigment accumulation. During light-independent anthocyanin biosynthesis, positive or negative transcription factors are activated or suppressed to form an MBW complex regulating the expression of anthocyanin biosynthetic genes.

However, some potato, turnip, and sweet potato varieties accumulate pigments as underground tubers, and it is obvious that the regulation of light-independent anthocyanin biosynthesis in these tubers is disintegrated in the HY5-COP1 signaling pathway. In addition, candidate HY5-independent regulators have been identified to regulate anthocyanin biosynthesis in tomatoes [136], which broadens our understanding of light-dependent and -independent anthocyanin biosynthesis. In flesh-colored tubers of the potato, cells lose pigment production when purple cells are continuously subcultured, turning from purple to white. StMYBATV, an anthocyanin repressor, may contribute to stopping anthocyanin biosynthesis in potato cell culture. Moreover, the level of DNA methylation is also associated with reprogramming the metabolism of anthocyanins [137]. In *Aft*/*Aft atv*/*atv* tomato plants, SlMYBATV loses its function to compete with SlAN2-like to interact with SlJAF13, which makes SlAN2-like interact with SlJAF13 and SlAN11 to form an MBW complex to activate the expression of SlAN1 and SlAN11 in a HY5-independent manner. Then, SlAN2-like interacts with SlAN1 and SlAN11 to regulate the transcription of anthocyanin biosynthetic genes to accumulate pigments in fully purple-skinned tomatoes [71]. Briefly, light signals induce anthocyanin accumulation via the HY5-mediated expression of anthocyanin biosynthetic genes. However, the regulation of light-independent anthocyanin biosynthesis is due to protein factors located upstream of the anthocyanin biosynthesis pathway to activate the expression of the MYB transcription factor and MBW components. The protein factors might be loss-functional repressors or HY5-independent activators (Figure 3).

## 5. Conclusions and Perspectives

Although much is now known about the regulatory pathway of anthocyanin biosynthesis, the functions of protein factors involved in anthocyanin accumulation in plants continue to be explored. In addition to the roles of the MBW complex and anthocyanin biosynthetic genes that determine spatial and temporal-specific pigmentation, there are other signal regulatory factors that respond to the environmental and developmental signals to activate the anthocyanin biosynthetic pathway. The specific mechanism by which anthocyanin can form in a light-dependent and -independent manner needs to be elucidated. Similarly, the signal factors related to abiotic stress such as low temperature, drought, and salinity-induced anthocyanin biosynthesis also remain to be identified. Moreover, epigenetic regulation, especially the methylation of protein-coding genes related to anthocyanin production in plants is still in its infancy. In addition, an integrated regulatory network of anthocyanin biosynthesis controlled by transcription factors of development and abiotic-response factors needs to be further explored. Overall, with the research progress of genetics and molecular biology, a clearer understanding of the mechanism of anthocyanin biosynthesis and accumulation will be obtained and provide a theoretical and practical basis for crop breeding in the future.

## Figures and Tables

**Table 1:** Chemical structure and physical characters of the six common anthocyanindins.

Chemical Structure	Substitution		Anthocyanidin Name	Color	λmax in the Visible Spectrum
	R1	R2			
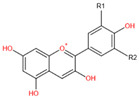	H	H	Pelargonidin (Pg)	Orange	494 nm
	OH	H	Cyanidin (Cy)	Orange-red	504 nm
	OH	OH	Delphinidin (Dp)	Blue-red	508 nm
	OCH3	H	Peonidin (Pn)	Orange-red	506 nm
	OCH3	OH	Petunidin (Pt)	Blue-Red	508 nm
	OCH3	OCH3	Malvidin (Mv)	Blue-red	510 nm
